# Notes from London

**Published:** 1897-09

**Authors:** J. L. Campbell

**Affiliations:** Atlanta, Ga.; London


### NOTES FROM LONDON.

LONDON, August 13, 1897.

We have had another case of carcinoma of the rectum on which Mr. Frederick Eve did a Kraske operation with splendid results. The growth extended from about an inch and a half above the anus to the sigmoid flexure, so the rectum had to be cut off so low down he thought best not to try to unite it. He therefore removed the coccyx and the sacrum to the third posterior sacral foramen and brought the colon out at that point, making an artificial anus. The patient did well, and is up and about the ward now.

We have had two cases of filaria sanguinis hominis. The first was a negro from the West Indies. He had a large swelling in his left groin, just below Poupart's ligament. This lump was about the size of a new-born baby's head, or a little larger. It was thought at first that it was a lipoma, as it resembled one very much and no fluctuation could be obtained. He was sent to the Royal College of Surgeons, and there a gentleman who had been in the West Indies for some time saw him and recognized the lump as one very common among the natives, and due to plugging of the lymph vessels by embryos of the filaria. His blood was then examined and found teeming with embryonic filaria. They were present in the superficial circulation only at night after 10 o'clock, and I will say that they were very punctual as to time, for we have examined his blood between 9 and 10, but when we would wait only a few minutes past 10 they were always present. When the patient was operated on the tumor was found to contain a quantity of chyle covered or surrounded by fat. The parent worm was found in the tumor. There was also a tumor beginning in the right groin, wphich was removed.

The second case was a young man who had been in the employ of the cable company and been in Australia, India and China. He came in with a hydrocele of the left side, which, when it was tapped, was found to contain chyle. The presence of filaria was expected, and we found them in the fluid. His blood was also examined, and they were found, but like the others they were out only at night. This patient had on the right side a varicose-like enlargement along the cord which was removed, and filaria were found in the fluid squeezed from the mass which was enlarged lymphatics.

The parent worm, which was found present in the isolated lymph glands of the first case, was about two and one-half inches long and very slender. The embryos found in the blood are about three-tenths of a millimeter long and about the diameter of a red blood corpuscle. When stained they appear to be composed of cells irregularly distributed, but more numerous at either extremity. They are very active when in blood freshly drawn from the finger, and live under the cover-glass for twenty-four to thirty hours. We have not been able to discover the remains of the embryonic capsule described by some. The parent worm usually inhabits the thoracic duct and gives off thousands of these embryos, which soon burst their capsule and appear in the blood. It is supposed that they are conveyed from the blood of man by the mosquito, which in sucking the blood gets a few embryo with it, and they undergo some change in the body of the mosquito or in the water in which the mosquito may fall, and when a man drinks this water the filaria is ready to develop a parent worm.

When the lymph vessels are blocked by these embryos they enlarge, and if not sufficiently protected by elastic tissue rupture, and abdomen, causing chyluracites; or, as in the case we had, into the abdomen, causing chyluracites; or as in the case we had, into the tunica vaginalis, causing chylous hydrocele.

An interesting case of Jacksonian epilepsy has just been discharged—a woman who was in the hospital twice in 1892, under Dr. Stephen MacKenzie. She was having fits at that time, and as she had a syphilitic history, it was diagnosed gumma of the brain. She improved on mercury and iodide, and was, except at a few intervals, free from fits until she came in last May. The fits were much worse and more frequent, and she was having headaches—at that time she was having about thirty fits a day. They lasted twenty or thirty seconds. Her eyes were drawn to the left and fixed, then her head was drawn slowly to the left, then contractions of the left eyelid extending to the whole side of the face, left shoulder and arm. The legs were involved only slightly, the contractions ending in the right shoulder. There was also paresis of the left side between the fits, which increased to paralysis. There had been marked optic neuritis during her previous stay in the hospital, but none at the present. The knee-jerks were present, exaggerated on the left. The fits increased in number so that she had from May 11th to 16th, seven hundred fits. At that time she was transferred to the surgical side. Her head had been shaved and the fissure of Rolando (right side) had been marked out. Mr. Eve trephined over the middle of the fissure, making an opening about an inch and a half in diameter. The brain bulged and did not pulsate. The opening was enlarged. The dura mater was opened, but nothing could be seen. He then explored the Rolandic area with his finger, but found nothing except a few adhesions nearer the superior longitudinal sinus. He also introduced a trocar in three places, but found nothing except a little softening. Some of this was removed with a spoon, and the dura closed with silk and the scalp wound with silkworm gut. The patient came out from the chloroform nicely, but continued to have fits. But she had some intervals of two or three hours at a time when she would not have a fit. She was perfectly conscious, and was able to talk rationally until a fit would interrupt her, then she would resume the conversation. The fits involved the right side as well as the left, after the operation, and at times the contractions were very severe. They gradually grew less, both in number and severity, and she was entirely free from them for a month, when she was discharged. She had regained the use of her hand and leg and was able to walk. She was, however, unable to locate the point at which she was touched on the left side, especially the forearm. She had been given iodide of soda, and bromide of ammonia since the operation, so Mr. Eve said he was not sure which did the work, the operation or the medicine, but as she had had iodide before, he supposed the operation did some good. She had over three thousand fits while she was in the hospital.

J. L. CAMPBELL, M.D.
(*Atlanta, Ga.*)

